# European landrace diversity for common bean biofortification: a genome-wide association study

**DOI:** 10.1038/s41598-020-76417-3

**Published:** 2020-11-13

**Authors:** Leonardo Caproni, Lorenzo Raggi, Elise F. Talsma, Peter Wenzl, Valeria Negri

**Affiliations:** 1 Dipartimento di Scienze Agrarie, Alimentari e Ambientali (DSA3), Università Degli Studi Di Perugia, Borgo XX Giugno 74, 06126 Perugia, Italy; 2 Division of Human Nutrition and Health, Wageningen University and Research, PO Box 17, 6700 AA Wageningen, The Netherlands; 3 HarvestPlus, International Center for Tropical Agriculture (CIAT), Km 17 Recta Cali-Palmira, Cali, Colombia; 4 Genetic Resources Program, International Center for Tropical Agriculture (CIAT), Km 17 Recta Cali-Palmira, Cali, Colombia

**Keywords:** Agricultural genetics, Genetic association study, Plant genetics

## Abstract

Mineral deficiencies represent a global challenge that needs to be urgently addressed. An adequate intake of iron and zinc results in a balanced diet that reduces chances of impairment of many metabolic processes that can lead to clinical consequences. In plants, bioavailability of such nutrients is reduced by presence of compounds such as phytic acid, that can chelate minerals and reduce their absorption. Biofortification of common bean (*Phaseolus vulgaris* L.) represents an important strategy to reduce mineral deficiencies, especially in areas of the world where this crop plays a key role in the diet. In this study, a panel of diversity encompassing 192 homozygous genotypes, was screened for iron, zinc and phytate seed content. Results indicate a broad variation of these traits and allowed the identification of accessions reasonably carrying favourable trait combinations. A significant association between zinc seed content and some molecular SNP markers co-located on the common bean Pv01 chromosome was detected by means of genome-wide association analysis. The gene *Phvul001G233500*, encoding for an E3 ubiquitin-protein ligase, is proposed to explain detected associations. This result represents a preliminary evidence that can foster future research aiming at understanding the genetic mechanisms behind zinc accumulation in beans.

## Introduction

Micronutrient deficiencies globally afflict more than two billion people^1^. Such deficiencies mostly occur when the intake of minerals and vitamins is too low to maintain good health and development or can also be caused by a combination of factors such as poor diet, disease and/or increased micronutrient needs^2–4^. Limited access, low availability, scarce intake or complete lack of fisheries and livestock in the diet, consumption of staple crops characterised by low tissue mineral concentration, and/or low mineral bioavailability can result in the impairment of many metabolic processes leading to clinical consequences^5–7^. In this context, mineral deficiencies are currently considered among the most serious global challenges to face in the next decades^6^.

Among pathologies caused by mineral deficiencies, anaemia affects roughly a third of the world’s population. The World Health Organisation assessed that worldwide 42% of pregnant women, 30% of preschool children (aged 0 to 5 years) and 12.7% of men older than 15 years are anaemic^8^. It has been recently estimated that roughly half of the cases of anaemia worldwide are associated with a scarce iron dietary intake and absorption^9,10^. Iron is an essential component of haemoglobin in red blood cells and myoglobin in muscles, which contain around 60% of total body iron^10^. In humans, iron is necessary for correct functioning of several cellular mechanisms, including DNA synthesis and mitochondrial energy generation^11^. Indeed, Iron Deficiency Anaemia (IDA) has been associated with several chronic diseases such as chronic kidney disease^9^, chronic heart failure^12^, cancer and inflammatory bowel disease^13^.

Zinc is the second most abundant mineral element in the human body and is the most abundant intracellular one^14^. Although its abundance, in humans there is no dedicated store; the lack of a zinc reserve causes impairment of some physiological functions when the availability of this element is scarce. It has been reported that inadequate zinc intake—as result of a diet low in zinc itself or rich in phytate—is the main cause of zinc deficiency^15,16^. As zinc has a large number of physiological roles in humans, especially in regulating growth and functioning of the immune system^17^, a deficiency can lead to diverse clinical conditions among which growth failure, easy development of infections or decline in muscle work capacity can have the most severe consequences^15^. Regarding zinc, its deficiency seems to be as common as IDA^18^.

Many crops—including legumes, cereals and vegetables—are rich sources of phytic acid (myo-inisitol-1,2,3,4,5,6-hexa-kis-phospate, InsP6) and its derivatives (InsP5, InsP4)^10,16^. IndP6 and its derivates are the most abundant storage form of phosphorus in plant seeds; they are accumulated during the ripening period and constitute 60% to 90% of total phosphorus store in seeds^19^. Converted into phosphates during germination, these compounds are the major source of phosphorus during seedling development^20,21^. On the other hand, due to their ability to chelate minerals, these compounds act as anti-nutrients: phytic acid binds to mono and divalent dietary mineral cations forming phytate complexes that are very stable at neutral pH^22,23^. A high phytate intake is generally associated with reduced mineral absorption—especially of iron and zinc—in the gastrointestinal tract^16^. Iron and zinc bioavailability for humans is generally estimated through molar ratios calculation of phytate:minerals. It has been reported that molar ratio values of phytate:iron above 1 have negative effect on iron absorption^24^ while a phytate:zinc molar ratio major than 15 is associate with low zinc bioavailability^25^.

In order overcome mineral deficiencies, several strategies can be considered: (i) food diversification, (ii) mineral supplementation, (iii) food fortification and (iv) biofortification^2,7^. The latter is defined as a process that relies on agronomical practices, conventional plant breeding or modern biotechnology to increase nutrient density and bioavailability in edible parts of crops^2^. Even if many nutritional traits can possibly be enhanced through biofortification, to date, most of research efforts focused their attention on minerals and vitamins^26^.

Common bean (*Phaseolus vulgaris* L.) is one of the most important sources of proteins and nutrients, like iron and zinc, for millions of people living in areas of the world where prevalence of undernourishment is the highest (e.g. Eastern Africa, as part of the Sub-Saharan Africa (31.4%) and the Caribbean (16.5%))^1,27^. Indeed, in some parts of these regions, cereal/bean-based diets can cause iron and zinc deficiency^27^. As such, biofortification of beans would allow to increase dietary intake of iron and zinc without requiring any food behaviour change of people living in the above-mentioned areas, where beans have a key role in their diet, making this approach potentially successful.

It is well known that common bean offers wide variability in terms of iron and zinc concentration, which is a basic requirement for attaining biofortification through plant breeding.

In common bean iron concentration ranges from 35 to 90 µg/g^28^ and is higher when compared to other major crops such as rice (6.3 to 24.4 µg/g)^29^, wheat (25 µg/g to 56 µg/g)^30^ and maize (9.6 to 63.2 µg/g)^31^. This species also shows relatively high zinc seed content (21 to 54 µg/g^28^). Nevertheless, common bean seeds are also characterised by high phytate content (1–3% of total seed weight^16,32^) that significantly reduces mineral bioavailability.

The common bean is a predominantly self-pollinating species, characterised by a diploid set of 11 chromosomes. The germplasm of this species can be classified into two distinct genepools; many studies confirmed such genetic structure that derives from two independent domestication events, occurred in Central and South America, giving rise to the Mesoamerican and the Andean genepool, respectively^33^.

Screening unexplored common bean diversity can possibly disclose accessions of interest for breeding and, at the same time, allows to study which genetic determinants can potentially contribute to the regulation of various traits, including those relevant for biofortification.

To date, genetic control of iron, zinc and phytate seed content in beans has been the object of several studies. In this species, Quantitative Trait Loci (QTL) analyses of biofortification-related traits have been conducted using bi-parental mapping populations from both intra and inter genepool crosses^34–36^. Some genomic regions were already associated with the accumulation of iron and zinc in seeds, most notably in 8 out of 11 chromosomes^37^; a similar approach has also been used for phytate. It has been shown that a number of QTLs control the accumulation of phytate and phosphorus in bean seeds. Some of these QTLs were associated with phytic acid pathway genes, in particular the *myo-inisitol (3)P1 synthase* gene as well as other loci in the bean genome^38^.

Currently, Genome Wide Association Studies (GWAS) are regarded as the next step, after QTL mapping, to potentially identify candidate genes involved in the control quantitative traits^39^. The GWAS approach represents an alternative strategy to QTL mapping, in fact it can generally consider a higher number of recombination events by using a wide panel of diverse individuals within a defined species^40^, each of them potentially characterised by a diverse “recombination history”. GWAS has been applied to a multitude of quantitative traits in common bean^41–44^, including those related to iron and zinc biofortification^37,45^.

During the last decade, progress has been made to foster biofortification of staple crops^46^; in fact, many reliable and cost-effective methods have been developed and validated to screen large panels of accessions. Regarding minerals, methods based on Energy Dispersive X-Ray Fluorescence (EDXRF) technology have been developed for wheat, rice, pearl millet^47^ and, recently, also for maize and common bean^48^. The EDXRF is based on the principle that each element upon exposure to X-rays of suitable energy produces secondary ‘fluorescent’ X-rays. The emitted X-ray spectrum is indicative of the element and the intensity is related to its concentration^48^. The EDXRF is a non-destructive technology (i.e. no chemical reagents are needed), a feature that reduces costs, time and avoids the production of toxic waste.

The aims of this work were to: (i) explore the diversity of a panel of highly homozygous common bean genotypes for iron, zinc and phytate seed content, (ii) use reliable and cost-effective methods for phenotyping, (iii) identify genotypes characterised by high biofortification potential and (iv) test associations between the studied quantitative traits and the genetic constitution of the panel.

## Results

### Iron, zinc and phytate quantification method evaluations

Considering the high number of analysed samples, the quick, non-destructive and, chemical reagent-free EDXRF method was the one of choice for iron and zinc analyses. Low coefficients of variation between experimental replicates, recorded for both minerals (0.9% and 2.3% for iron and zinc, respectively), indicated high reproducibility of the used protocol.

To ensure a high quality phytate characterisation, a method based on the procedure developed by Latta and Eskin^49^ was used after minor modification. With an average coefficient of variation of total seed phytate between experimental replicates of 2.9% only, this method showed high reproducibility too.

### Iron, zinc, phytate quantification and data analysis

Iron, zinc and phytate quantification was performed on seed samples from a single, partially replicated experiment carried out under controlled conditions; the collected samples allowed to successfully characterise 93% of the 192 genotypes included in the trial. Results of the analysis for possible spatial biases due to plant position showed that the Completely randomised design (Crd) was always the most efficient model over the others; this evidence confirmed that the used experimental design did not introduce any spatial bias. Best Linear Unbiased Estimators (BLUEs) were then calculated for the three traits (Table S1). BLUEs analyses showed that the panel is characterised by a high level of phenotypic diversity (Table 1). Estimated levels of broad sense heritability (He^2^_B_) were also relatively high for zinc and phytate while lower for iron (Table 1).

**Table 1:** Descriptive statistics, *He*^*2*^_*B*_ and spatial model along with its efficiency of iron, zinc and phytate seed content BLUEs of 192 common bean genotypes.

	Mean	Range (min–max)	CV (%)	He^2^_B_^a^ (SE)	Spatial model	Efficiency (%)^b^
Iron (µg/g)	61.7	38.4–93.7	31.3	0.168 (0.148)	Crd^c^	100
Zinc (µg/g)	30.7	18.9–43.6	31.1	0.414 (0.131)	Crd	100
Phytate (mg/g)	11.9	4.8–19.9	34.7	0.434 (0.133)	Crd	100

^a^Broad sense heritability.

^b^Spatial model efficiency.

^c^Completely randomised design.

A significant correlation between ‘iron and zinc’ seed content was observed (Pearson’s coefficient 0.50, *p* ≤ 0.001); significant correlations were also observed between ‘phytate and iron’ and ‘phytate and zinc’ with values of 0.38 and 0.46, respectively (*p* ≤ 0.001). Within the screened panel, iron and zinc seed content showed a wide range of variation (Table 1) while phytate was characterised by lower range of variation (Table 1).

Three genotypes of Mesoamerican origin showed particularly high iron seed content (> 90 µg/g; 95th percentile = 82.9 µg/g): two of the genotypes derived from different landrace climbing accessions from Costa Rica (PI-309837 and PI-309885) while the other from an Italian bushy landrace (4135, FAO-363). Genotypes 4914, 7185, G993, G20110A, PI-309837, PHA1916 and PHA1474, derived from six landraces and a single cultivar, resulted the most promising accessions for zinc seed content (95th percentile = 41.8 µg/g). Finally, five accessions characterised by low phytate were also identified: developed from an Italian landrace of Andean origin, genotype 5071 showed the lowest value (≤ 6.0 mg/g, unique case) while other four genotypes—PHA6857, G1018, G10252, G11573—showed a phytate content minor than 7.0 mg/g (5th percentile = 7.2 µg/g).

**Figure 1:**
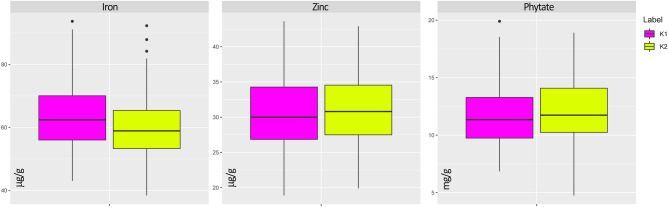
Boxplots of BLUE values of 178 common bean genotypes according to their genepool attribution: Mesoamerican (K1, magenta) and Andean (K2, yellow).

Results of the characterisation were also analysed considering genotype attributions to the Mesoamerican and the Andean genepool by taking advantage of a previous analysis of the genetic structure of this panel^41,50^. When the factor ‘genotype origin’ (i.e. Mesoamerican or Andean) was considered, no significant differences (*p* ≤ 0.001) were observed (Fig. 1). Dispersions of iron, zinc and phytate BLUEs—grouped according to the genepool attribution—are reported in Fig. 1.

**Figure 2:**
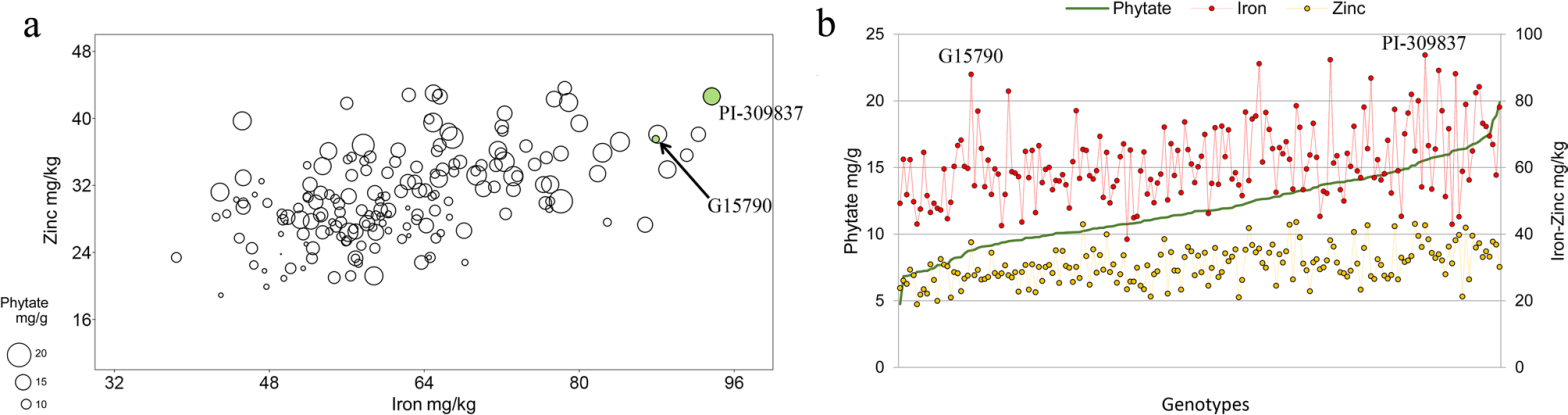
Bubble plot (**a**) and line plot (**b**) of iron, zinc and phytate BLUEs of 178 common bean genotypes. In the bubble plot (**a**), the two axes show iron and zinc seed content, while the area of the bubbles is according to total phytate seed content. Genotypes with best combination of the three traits are highlighted in green. In the line plot (**b**), genotypes are plotted according to increasing levels of phytate (green dots), for each genotype, iron (red dots) and zinc (yellow dots) seed content are also reported.

Bearing in mind the desired levels of the three traits together (i.e. high iron, high zinc and low phytate), the pure line G15790—derived from a Spanish landrace of Andean origin—resulted the most promising: its seed is characterised by high iron (87.9 µg/g), high zinc (37.5 µg/g) and, low phytate content (8.8 mg/g) (Fig. 2a,b). Furthermore, the pure line PI-309837—derived from a Mesoamerican landrace from Costa Rica—showed the best combination of iron and zinc, both over the 95th percentile (93.7 and 42.6 µg/g, respectively); however, this line is also characterised by high phytate seed content (15.6 mg/g) (Fig. 2a,b).

**Figure 3:**
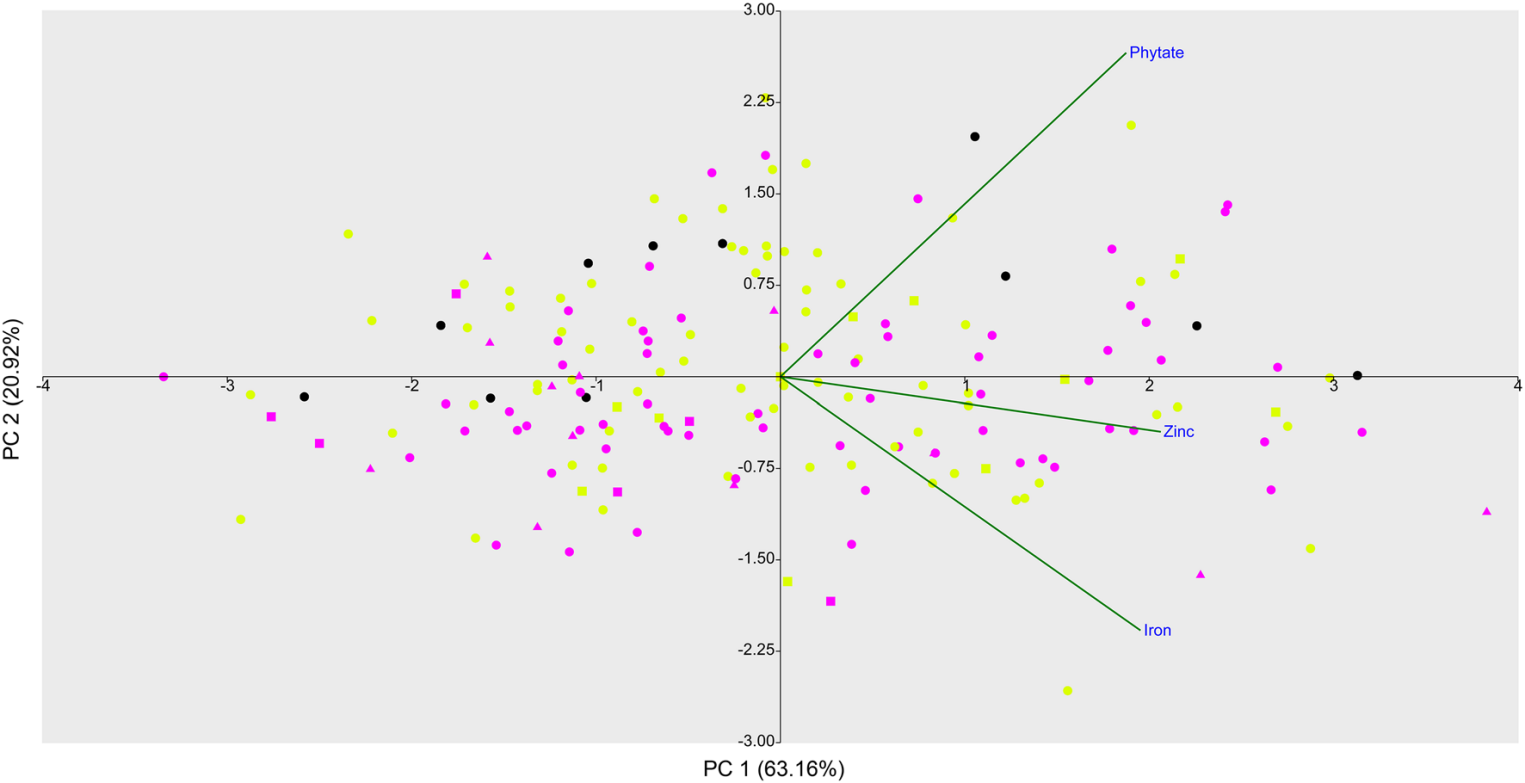
Two-dimensional PCA of iron, zinc and phytate BLUEs of 178 common bean genotypes. In the biplot genotype colours are according to genetic STRUCTURE groups: K1 Mesoamerican (magenta), K2 Andean (yellow) and admixed (black) while shapes to geographical origin: Europe (dot), Central America (triangle) and South America (square). In the biplot, projections of the original axes (variables) onto the scattergram are reported in green.

In the Principal Component Analysis (PCA) of the three traits, the first two principal components explained 84.1% of the total variation (Fig. 3). According to the results, neither geographical origin (i.e. Europe, South and Central America) nor membership to the different genetic groups (Mesoamerican or Andean) explained the observed phenotypic diversity. As from results of the PCA, most of the samples are characterised by low iron, zinc and phytate content (PC1, negative values).

### Estimation of iron and zinc relative bioavailability

Within the screened bean diversity panel, phytate:iron ranged from 8.2 to 32.0 (mean 16.7) while phytate:zinc from 19.8 to 76.5 (mean 39.0) (Table S1). Results indicated genotype 5071 (ITA-363) and G15790 (CIAT) as those characterised by the best molar ratios with values of 8.2 and 8.4 and of 19.8 and 23.2 for phytate:iron and phytate:zinc, respectively. Notably, these two genotypes were already identified for lowest phytate seed content (5071) and best overall combination of the three traits (G15790).

### Genome-wide association analysis

**Figure 4:**
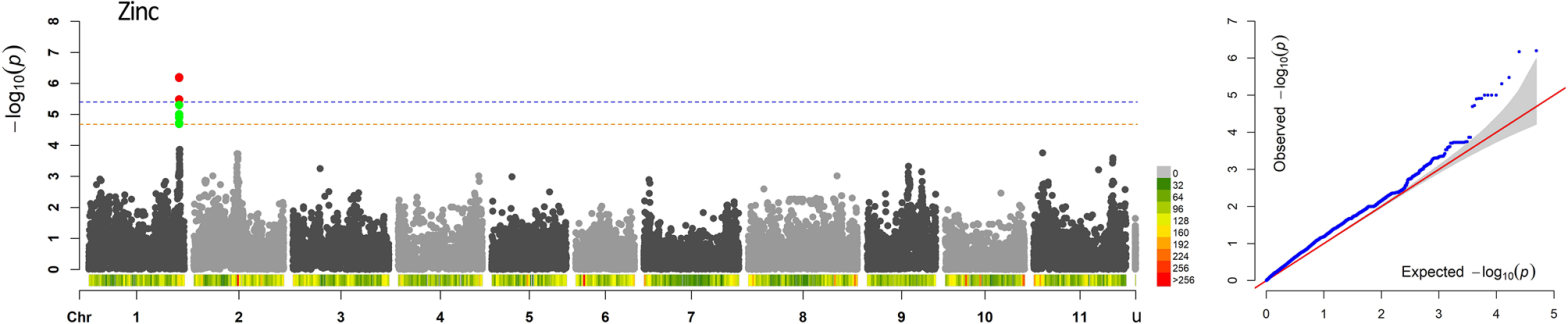
Manhattan and QQ-plot of zinc seed content. In the Manhattan plot, the horizontal lines indicate the genome-wide significance thresholds: 5.4 (blue, Bonferroni correction based on α = 0.01) and 4.7 (orange, based on α = 0.05). SNPs with a *p*-value above the selected thresholds are highlighted in red and green, respectively. For each chromosome, SNP density within 1 Mb window is reported. SNP density is according to the colour key.

In the GWAS analysis—carried out through a Mixed-Linear Model (MLM) accounting for both population structure and genetic cryptic relatedness on a pruned-dataset of 49,518 SNPs—two thresholds of significance were calculated at two different alpha levels. Since 2443 independent recombination blocks were identified within the panel, the two Bonferroni thresholds of significance for association analyses were set at 5.4 and 4.7 – log_10_(*p*) using alpha values of 0.01 and 0.05, respectively. Considering such conservative thresholds, no significant associations were detected for iron and phytate seed content. However, the GWAS analysis revealed significant associations between zinc seed content and 12 SNPs located on a distal portion of chromosome Pv01, spanning over a window of about 120 kb (Fig. 4). The three SNPs characterised by lowest *p*-values (17648_70, 17648_54 and 17649_82) co-localised in a chromosome segment of 150 bp only and resulted significant also after the application of the most severe Bonferroni correction (alpha = 0.01). Significant markers within this genomic region explained a proportion of the phenotypic variation of zinc seed content from 0.136 to 0.153 (Table 2). Features of the three SNPs associated with zinc are reported in Table 2 while of the other SNPs significantly associated after the correction based on alpha = 0.05 in Table S2. The search of positional candidate genes for the detected associations resulted in the identification of *Phvul001G233500*. Such candidate encodes for a E3 ubiquitin-protein ligase characterised by a specific domain selectively interacting with zinc ions.

**Table 2:** List of SNPs associated with zinc seed content (α = 0.01).

SNP	Chromosome	Position^a^	−Log_10_(*p*)	R^2^	MAF
17648_70	Pv01	49,373,158	6.20	0.153	A (0.48)
17648_54	Pv01	49,373,142	6.17	0.153	A (0.48)
17649_82	Pv01	49,373,009	5.48	0.136	A (0.48)

Chromosome physical position, significance level (−Log_10_(*p*)) of the detected association, phenotypic variation explained by the SNP (R^2^) and Minor Allele Frequency (MAF) are reported.

^a^*P. vulgaris* reference genome v1.0.

### Linkage disequilibrium analysis

**Figure 5:**
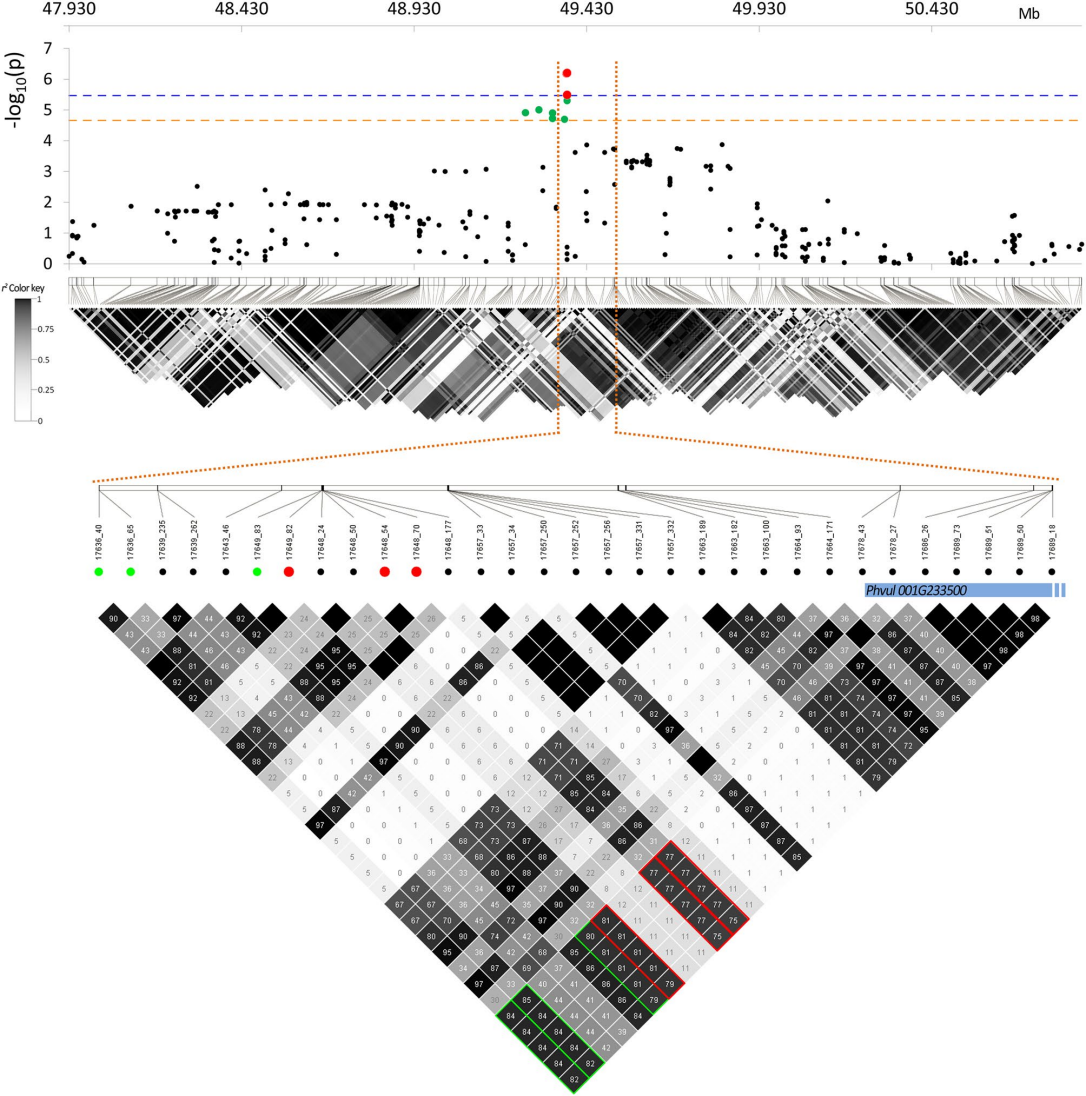
Top: Manhattan plot and LD heatmap of zinc seed content over a chromosomic region (Pv01) of ± 1.5 Mb centred on the associated SNP markers. In the Manhattan plot, the horizontal lines indicate the genome-wide significance thresholds: 5.4 (blue) and 4.7 (orange) for Bonferroni correction based on α = 0.01 and α = 0.05, respectively. SNPs with a *p*-value above the selected thresholds are highlighted in red and green, respectively. Bottom: LD heatmap zoom over a chromosomic region of 180 kb containing the associated SNPs and the positional candidate gene. In the figure *r*^*2*^ values (%) between SNP markers and the candidate are highlighted according to their significance: in red and green for α = 0.01 and α = 0.05, respectively. In the figure physical position of the identified gene (*Phvul001G233500*) is reported (blue bar).

According to our results, the three SNP markers characterised by the lowest *p*-values and the candidate gene fall in a genomic region of about 200 kb (Fig. 5). Size of this genomic region is compliant with the evidence from a previous study—carried on the same panel of genotypes and using the same SNP pruned-dataset—where LD decays on average within 240 kb^41^.

Results of the Linkage Disequilibrium (LD) analysis showed that SNP markers significantly associated with zinc seed content and the identified positional candidate *Phvul001G233500* are characterised by high *r*^*2*^ values (Fig. 5). In particular, the three most significant markers are characterised by *r*^*2*^ values ranging from 0.75 to 0.81 with markers localised within the gene (Fig. 5, bottom, red dots); such evidence shows non-random occurrence of alleles at significant loci with those located within the sequence of best identified candidate gene. This also applies to markers that remained significant after the application of Bonferroni correction considering an alpha of 0.05 (4.7) (Fig. 5, bottom, green dots).

## Discussion

The used common bean panel of diversity^41,50^ resulted an excellent experimental material to screen the within-species phenotypic variation of iron, zinc and phytate seed content. The methods used for the phenotyping showed high reproducibility and, at the same time, can be regarded as cost-effective tools for biofortification-oriented breeding programmes. According to our results, the explored phenotypic diversity was high for all three traits when compared to other extensive germplasm characterisations^26,38,45,51^. Results of our screening allowed to identify promising accessions carrying favourable levels or combinations of the studied traits. In addition, taking advantage of the latest genomic analysis methods, we were also able to detect a candidate potentially involved in the regulation of zinc accumulation in bean seeds.

### Iron, zinc and phytate quantification methods

Regarding the available options to perform simultaneous determination of mineral elements in plants, Inductively Coupled Plasma-Optical Emission Spectrometry (ICP-OES) has been extensively used in recent years^52^ and applied to biofortification-oriented research^51,53^. Even if ICP-OES has proved to be an accurate method, it requires lengthy sample preparation, costly reagents and highly trained analysts; consequently, it does not provide as a matter of fact a practical and cost-effective tool to perform extensive germplasm characterisation. On the other hand, EDXRF has shown to be a cost-effective method to screen high number of samples being already validated for iron and zinc in common bean, maize and cowpea^48^. In our study this technique has been successfully used to generate an accurate and reproducible phenotypic characterisation.

Regarding the quantification of phytate seed content, the use of the modified Latta-Eskin method allowed to produce an extensive phenotypic characterisation too. Low cost of reagents, and the use of a resin that can easily be regenerated multiple times, makes this method cost-effective and suitable to screen within-species diversity. In addition, low coefficients of variation between replicates further confirmed its reproducibility and reliability. The combination of these methods opens new possibilities to effectively phenotype large common bean germplasm panels at low cost and high effectiveness; in our opinion, these aspects are relevant conditions to enhance future efforts of biofortification-oriented common bean breeding programs.

### Iron, zinc and, phytate characterization

Results of the phenotypic characterisation of iron and zinc—based on a single, partially replicated experiment under controlled conditions—are in line with those reported by other studies^28,54,55^. In particular, we observed iron and zinc ranges compliant with levels recorded in the world largest common bean core collection (1031 accessions) held at CIAT^28^, where iron ranged from 34 to 89 µg/g (mean 55 µg/g) while zinc from 21 to 54 µg/g (mean 35 µg/g). Concerning phytate, our screening revealed the existence of high variability; reviewing the existing literature, Sparvoli and colleagues reported a wider window of variation (from 3.4 to 28.7 mg/g)^16^; however, it should be noted that the reviewed studies used different quantification methods and germplasm, this could have affected the reported variability.

The three genotypes identified in our study that showed the highest potential to increase iron seed content were developed starting from Mesoamerican landrace accessions. Even if no correlation between geographic distribution and iron seed content has been reported in the literature, and confirmed by our data, a previous study^28^ highlighted the tendency of Andean genotypes to have higher iron concentration than Mesoamericans. On the contrary, in our diversity panel, Mesoamerican genotypes were characterised by a slightly higher average value when compared to the Andeans (64.1 µg/g vs. 59.6 µg/g, respectively); however, this difference was not significant indicating that, in this species, iron seed content is not substantially affected by genepool membership. Similarly, the phenotypic characterisation of zinc allowed the identification of other genotypes that could be exploited to develop cultivars characterised by an increased content of this mineral. Also in this case, genepool membership did not affected the expression of this the trait. Interestingly, characterising a common bean collection of breeding lines of Middle American ancestry, McClean and colleagues observed iron content ranging from 47.0 to 83.5 (mean 61.4) while zinc from 23.50 to 38.1 (mean 30.87)^56^. Even if our collection encompasses a lower number of Mesoamerican genotypes, its variability for the same minerals resulted wider: from 43.0 to 93.7 (mean 63.61) for iron and from 18.9 to 43.6 (mean 30.4) for zinc. In our opinion this is a good indication that supports the use of landraces when screening complex quantitative traits like accumulation of iron and zinc in seeds.

### Iron and zinc relative bioavailability

The quantification of iron, zinc and phytate seed content allowed to estimate relative bioavailability of the two minerals that is commonly calculated as the molar ratio of phytate:minerals. Values of the molar ratios observed within the diversity panel gave a comprehensive overview of the natural variation of this important nutritional aspect within this species. In this regard, quite different conditions were found. Interesting enough, some accessions showed promising bioavailability levels, especially for zinc. In particular, the best identified genotype (5071, ITA 363) is characterised by a phytate:zinc molar ratio of 19.8 that is very close to the threshold of 15 generally associated with moderate zinc bioavailability (corresponding to about 15% of total zinc bioavailable)^25,57^. It is noteworthy to mention that the molar ratios reported in our study were obtained though analysis of unprocessed bean seeds. In fact, it has been showed that seed phytic acid content can decrease after seed processing (soaking in particular) due to activation of an endogenous phytase (myo-inositol hexakisphosphate phosphohydrolase)^19^. This suggests that higher mineral bioavailability levels are expected to occur after processing; this also applies to the most promising genotypes.

These findings confirm the importance of screening underutilised landrace germplasm when looking for quality traits. Often characterised by favourable combinations of quality traits (e.g. good taste or flavour) landraces also offer great potential to attain better human nutrition as shown by our results. For instance, the line derived from the accession G15790 showed good phytate:minerals molar ratio values and best overall combination of the three traits; this genotype can profitably be included in biofortification-oriented common bean breeding programmes or—after testing consumer acceptance—even be used for direct cultivation and human consumption.

### Genome-wide association analysis for biofortification

The use of a partially replicated experimental design allowed to perform association analyses using data from a single experiment carried out under controlled conditions allowing to test for possible spatial biases due to plant position. As from the results of the bi-dimensional spatial analysis, the ‘Completely randomised design’ (Crd) was the best model for BLUEs calculation showing that no biases affected the produced phenotypic dataset. This evidence confirms that use of same soil composition, soil volume and, growing conditions allowed to achieve high standardisation of the experiment. The partially replicated experimental design also allowed to estimate broad sense heritability of the three studied traits using data from single year; this is particularly relevant since a high heritability is a prerequisite to succeed GWAS^42,58^. We observed relatively high heritability for zinc and phytate while a lower value was estimated for iron. According to our results, iron seed content variability within biological replicates can be the main reason explaining the absence of associations with the genetic constitution of the diversity panel. However, it should be always considered that the reported heritability values are estimations based on a single experiment carried out in isolated and highly standardised conditions. Collecting data on phytate and mineral on the same genotypes in multiple locations across different growing seasons will allow to test the role of the environment on mineral and phytate seed accumulation.

The MLM used in this study, accounting for both genetic structure and kinship, allowed to identify significant associations between seed zinc content and a genomic segment in chromosome Pv01. According to our results *Phvul001G233500* is the best positional candidate to explain the effect of the significant SNPs co-located in this region, spanning over a chromosomic portion of 42 kb. *Phvul001G233500* encodes for a E3 ubiquitin-protein ligase which is an intracellular membrane-localised protein with E3 ligase activity and its C-terminus facing the cytoplasm. This protein is known to be involved in the regulation of Abscisic Acid (ABA) signalling. Encoded by the homolog *At3G55530*, the overexpression of this protein lead to an increased sensitivity to ABA in *Arabidopsis thaliana*, while loss of function alleles to a decreased sensitivity^59^.

More relevant for our study is the fact that this protein is characterised by a specific domain interacting selectively and non-covalently with zinc ions. In addition, in *P. vulgaris*, this gene is highly expressed in the green pods (i.e. during seed development); in our opinion, this evidence might explain its possible involvement in the translocation of zinc into seeds. Interestingly, a recent study by Izquierdo and colleagues^37^ evidenced the presence of a meta-QTL significantly associated with both iron and zinc seed content in the same region of chromosome Pv01. Even if the authors proposed a different candidate (i.e.* Phvul001G225000*), the genomic region harbouring this gene is relatively close to the one found in our work, at about 650 kb. Since in our study no clear association was found for iron seed content in the same chromosomic region, a potentially different QTL mechanism from that proposed by Izquierdo and colleagues emerges. However, results of both studies confirm that this distal region of chromosome Pv01 harbours genes involved in zinc accumulation; if additional studies will confirm the role of this genomic portion, this knowledge will contribute fostering future breeding activities aiming at releasing beans characterised by higher zinc content. It is also relevant to mention that another recent GWAS by Katuuramu and colleagues^45^ revealed the presence of other regions significantly associated with zinc content on chromosomes Pv07 and Pv10, not found in our study; these results were obtained screening cooked dry beans on a diversity panel composed of Andean genotypes only. The complex genetic control of the regulation of mineral content in seeds, together with the use of different germplasm can reasonably explain the identification of different chromosomic regions. Indeed, the amount of minerals in seeds depends on a high number of processes like their mobilisation from the soil, uptake by roots, translocation, and redistribution within the plant. Each of these processes is likely to be under the control of many genes, making the accumulation of minerals in seeds a highly complex polygenic trait^60^. It is known that the absorption and translocation of cations, such as zinc, is carried out by carbon-based compounds through non-covalent interactions^61^ as for the protein encoded by the gene identified in this study. This evidence supports a possible contribution or major effect of *Phvul001G225000* in the control of zinc accumulation in common bean seeds. Rather reliable in terms of number of analysed genotypes and SNP markers, minerals and phytate quantification methods and the GWAS model, this result must be considered a preliminary evidence; future GWAS, based on data from multilocation field trials, will possibly confirm the major role of this gene in the accumulation of zinc in bean seeds.

## Conclusions

Iron and zinc deficiencies are widely spread nutritional disorders, affecting billions of people worldwide. Thanks to many efforts of the international research community, iron and zinc biofortification has been already achieved for some crops where major genes involved in the translocation of these minerals have been identified^62–64^. Regarding the common bean, efforts are still needed to develop better iron and zinc biofortified varieties also characterised by high bioavailability of these minerals. Development and utilisation of biofortified bean varieties, characterised by high nutritional value, will potentially have a strong beneficial impact on more than two billion people living in areas where beans play a main role in their diet.

## Materials and methods

### Plant material

The bean diversity panel used in this study was already described by Caproni and colleagues^50^. Briefly, a total of 192 highly homozygous common bean genotypes (i.e. pure lines) were initially developed starting from 179 landraces and 13 cultivars by five generations of Single Seed Descent (SSD). The idea behind the constitution of this diversity panel was also to represent a relevant portion of the European common bean diversity (153 accessions) also including some accessions from the Americas (22 and 17 from South and Central America, respectively). A balanced representativeness of the two existing common bean genepools^65,66^ was obtained by including a similar number of Andean and Mesoamerican accessions in the panel^41,67^. The original accessions were obtained from the International Center for Tropical Agriculture (CIAT, Cali, Colombia) (70), University of Perugia (Perugia, Italy, ITA-363) (58), United States Department of Agriculture (USA) (34), IPK Gatersleben (Gatersleben, Germany) (28) and NordGen (Alnarp, Sweden) (2).

### Experimental setup

In order to produce seed samples for subsequent iron, zinc and phytate quantification, each genotype (entry) was grown under isolated condition in a nursery at the experimental station of the Department of Agricultural, Food and Environmental Science (DSA3) of the University of Perugia, located in Sant’Andrea d’Agliano (43°3′15.12″ N; 12°23′41.64″ E, 175 m a.s.l., Perugia, Italy), in a single experiment, carried out in 2017. Plant isolation was obtained covering the experiment with a standard anti-insect net. Plants were sowed in pots (diameter 40 cm), using a soil mixture of clay loam (40%) and potting compost (60%)^68^; plants were supplied with water throughout the growing cycle using an automatic drip irrigation system. Plants were arranged using a partially replicated randomised design in which five genotypes were replicated five times while two six times, producing a total of 222 samples out of 192 entries^41,50^.

### Sample preparation

At maturity, seed samples were manually harvested, dried, and stored carefully, avoiding any possible contact with metal objects. Seed samples were then prepared for the analyses as follows: (i) surface cleaned with ethanol to remove potential soil and dust traces; (ii) oven-dried at 60 °C for 72 h to standardise and reduce the relative humidity; (iii) 4.3 g of seeds (± 0.1 g) weighted and milled for 4.0 min in a custom-built system (CIAT, Cali, Colombia) equipped with elliptical chambered Teflon capsules and 10.0 mm zirconium oxide beads. The resulting fine flour was used to perform mineral and phytate quantification.

### Iron and zinc quantification

Iron and zinc analyses were carried out using EDXRF on an X-supreme 8000 (Oxford Instruments, Abingdon-on-Thames, UK) fitted with a 10-sample carousel. Scans were conducted on 4.0 g of sample in aluminium cups assembled with polypropylene inner cups, sealed at one end with 4 µm Poly-4 XRF sample film. Method calibration and validation are described in detail by Guild and colleagues^48^. Measurement conditions for the two minerals are reported in Table 3.

**Table 3:** EDXRF conditions used for the analysis of the samples.

Conditions	Iron	Zinc
Atmosphere	Air	Air
X-Ray tube	Tungsten	Tungsten
Voltage (kV)	26	15
Current (µA)	115	200
Acquisition time (s)	180	180
Tube filter	W5	A6

### Phytate extraction, purification, and quantification

Phytate extraction was carried out using 0.50 g of fine bean sample flour mixed with 20 ml of hydrochloric acid (HCl) 0.65 M in a 50 ml polypropylene tube (VWR, Radnor, Pennsylvania, USA). The resulting mixture was mechanically shaken using a VX 2500 multitube vortex (VWR, Radnor, Pennsylvania, USA) for 2 h and then centrifuged at 3600 rpm at 18 °C for 15 min in a 5810R centrifuge (Eppendorf, Hamburg, Germany). Then, an aliquot of 10 ml of the supernatant was transferred into a 15 ml polypropylene tube (VWR Radnor, Pennsylvania, USA).

Total phytate content was then quantified using a modified Latta and Eskin^49^ procedure, as in Park and colleagues^69^, using poly-prep pre-filled chromatographic columns containing AG-1-X8 anion exchange resin (100–200 mesh chloride form, 0.8 × 4 cm) (Bio-Rad Laboratories, Richmond, California, USA).

In order to bind phytates with the resin, 2 ml of the previously collected supernatant were diluted using 6 ml of deionised water (1:4) and loaded on the column placed on an Extraction Vacuum Manifold operating at atmospheric pressure. Column-bound phytate were washed using 10 ml of 0.07 M Sodium Chloride (NaCl) to eliminate free phosphates and other impurities. Phytate were subsequently eluted using 30 ml of 0.7 M NaCl (i.e. 3 × 10 ml washes).

Phytate quantification was carried out using an aliquot (900 µl) of the eluate mixed with 300 µl of the Wade reagent (0.03% iron (III) chloride, 0.3% sulfosalicylic acid). The absorbance of salicylate-Fe (III) was estimated at 500 nm using an Epoch 2 microplate spectrophotometer (BioTek, Winooski, Vermont, USA). For each experiment, the concentration of total phytate was estimated using a calibration curve prepared with a solution of phytic acid dipotassium salt (Sigma-Aldrich, Saint Louis, Missouri, USA) and 0.7 M NaCl, using concentrations ranging from 0 to 60 µg/ml. To monitor method performance, each sample was extracted, purified, and quantified in duplicate.

### Iron, zinc and phytate data analyses

The partially replicated experimental design allowed the calculation of Best Linear Unbiased Estimators (BLUEs) of iron, zinc and phytate seed content. BLUEs were generated using GenStat^70^ software^71,72^. In brief, for each of the three traits, BLUEs of genotype effects were calculated using the most suitable spatial model determined for the row and column field layout as described by Singh and colleagues^73^. Out of the nine tested models, the best was then selected in accordance to the Akaike Information Criterion^74^. This procedure also allowed the estimation of broad sense heritability (He^2^_B_) for each trait, based on data from a single, partially replicated experiment carried out under controlled conditions.

The following analyses were performed using the BLUEs dataset. Descriptive statistics, frequency distribution and Pearson’s correlation coefficients among traits were estimated using the R package ‘agricolae’^75^ and the software ‘Past3’^76^. A Principal Component Analysis (PCA) was then carried out on normalised data taking into account sample origin (Europe, Centrale and South America) and information on the genetic structure of the panel^41^. Trait differences between different genetic groups were then tested using the univariate t-test and visualised as data dispersions using the R package ‘ggplot2’^77^.

To identify genotypes characterised by high breeding potential for common bean biofortification, the three dimensions of data were jointly visualised in a bubble chart and in a scatterplot where samples were ordered by increasing phytate seed content. Finally, genotypes characterised by high iron and zinc (i.e. 95th percentile of iron and zinc seed content) and low phytate content (i.e. 5th percentile of total phytate seed content) were identified.

Relative bioavailability of iron and zinc was estimated through the calculation of molar ratios of phytate:iron and phytate:zinc, respectively^78,79^. The molar ratios were calculated using BLUEs of the three traits and a value of 660.3 g/mol as molecular weight of phytate as described by Castro-Alba and colleagues^57^.

### Genotyping

The genotyping was performed as described by Raggi and colleagues^41^. Briefly, DNA of the 192 genotypes was extracted using the TissueLyser II (Qiagen) and the DNeasy 96 plant kit (Qiagen, Hilden, Germany)^80^. Sample information is available at the National Center for Biotechnology Information. Before library preparation, concentration and quality of DNA were estimated by means of: (i) UV–vis spectroscopy with a Nanodrop 2000 (Thermo Scientific Waltham, Massachusetts, USA) and (ii) a fluorometric assay using a Qubit 2.0 (Invitrogen, Carlsbad, California, USA). A double digest Restriction-site Associated DNA sequencing (ddRAD-seq)^81^ approach was used for genotyping. Digestion was carried out using SphI and MboI restriction enzymes. The sequencing was performed on an Illumina HiSeq2500 platform (Illumina, San Diego, California, USA). The sequenced fragments were then aligned against the reference genome v. 1.0 using BWA-MEM^82^ and SNPs were called using functions implemented in Stacks 2.0^83^. The genotyping resulted in a dataset of 106,072 polymorphic loci. A pruned-dataset of 49,518 SNPs was obtained after removing loci and genotypes characterised by ≥ 10% missing data and loci with a minor allele frequency ≤ 5% and heterozygosity ≥ 2% using functions implemented in PLINK v. 1.07^84^ and Tassel v. 5.2^85^ software.

### Genome-wide association analysis and candidate gene identification

The association analysis was performed using the pruned-dataset of 49,518 SNPs trough a Mixed-Linear Model (MLM) implemented in Tassel v. 5.2^85^. The tested phenotypes consisted in the BLUEs dataset obtained from a single experiment carried out under controlled conditions. The MLM accounted for both population structure and genotype cryptic relatedness (kinship). In this regard, results of STRUCTURE and kinship analyses were retrieved from Raggi and colleagues^41^. Briefly, STRUCTURE results indicated that the common bean panel of diversity consists of two main clusters: 87 genotypes were attributed to K1 (Mesoamerican), 94 to K2 (Andean) while 11 resulted admixed. The kinship analysis showed different relatedness levels among genotypes and highlighted no genetic redundancy. Results of the association analysis were corrected for multiple testing using the Bonferroni adjustment considering two alpha values (0.01 and 0.05); the correction was applied considering the number of haplotype blocks within the panel, as described by Gabriel and colleagues^86^; number of haplotype blocks was inferred using a function implemented in PLINK software^84^.

Candidate gene search was carried out based on proximity to significant SNPs by browsing the *P. vulgaris* genome v. 2.1 in Phytozome 12.1^87^ as follows: sequenced fragments containing significant SNPs identified after GWAS analysis were aligned against the reference v. 2.1. Sequences of the putative candidate genes, translated into the corresponding protein, were then compared against the *A. thaliana* protein database (Araport11 protein sequences) using the online tool BLASTP in TAIR (https://www.arabidopsis.org/Blast/). Once the gene was selected, it was subsequently positioned on the common bean reference genome v 1.0 as the SNP dataset used in this study has been mapped using the same reference.

### Linkage disequilibrium analysis

To ascertain whether the identified SNP markers located in non-coding regions and candidate genes are in Linkage Disequilibrium (LD)—meaning that they tend to be inherited together—a LD analysis was carried out in HaploView 4.2^88^. Pairwise LD between markers (*r*^*2*^) was calculated within a window of ± 1.5 Mb around the most significant marker associated with the corresponding trait. To better visualise LD patterns between a candidate gene and the associated markers, further analyses were performed and visualised in narrower windows.

## Supplementary information

Supplementary Information.

## Data Availability

*Accession codes* NCBI, from SAMN12035168 to SAMN12035359. The genotyping dataset is available at: https://www.ebi.ac.uk/ena/data/view/PRJEB33063.
