# Supplementary material for: European landrace diversity for common bean biofortification: a genome-wide association study
Source: Sci Rep. 2020 Nov 13;10:19775. doi: 10.1038/s41598-020-76417-3 (PMC7666124; doi:10.1038/s41598-020-76417-3)
Supplement: Supplementary file 1 — Supplementary Information. [file 41598_2020_76417_MOESM1_ESM.pdf]

European Landrace Diversity for Common Bean Biofortification: A Genome-Wide Association Study

Leonardo Caproni[1,5], Lorenzo Raggi[1,5], Elise F. Talsma[2,3], Peter Wenzl[4], and Valeria Negri[1*]

[1] Università degli Studi di Perugia, Dipartimento di Scienze Agrarie, Alimentari e Ambientali (DSA3), Borgo XX Giugno 74, 06126, Perugia, Italy
[2] Wageningen University and Research, Division of Human Nutrition and Health, PO box 17, 6700 AA Wageningen, The Netherlands
[3] International Center for Tropical Agriculture (CIAT), HarvestPlus, Km 17 Recta Cali-Palmira,Cali, Colombia
[4] International Center for Tropical Agriculture (CIAT), Genetic Resources Program, Km 17 Recta Cali-Palmira, Cali, Colombia
These authors contributed equally

Table S1: BLUEs of iron, zinc and phytate seed content and phytate:iron and phytate:zinc molar ratios of 192 common bean (*P. vulgaris* L.) genotypes.

Accesion number	GENOTYPE code	STRUCTURE ASSIGNATION	Iron (µg/g)	Zinc (µg/g)	Phytate (mg/g)	Phy:Fe	Phy:Zn
CIAT_G13936	Pv_001	K2	NA	NA	NA	NA	NA
CIAT_G15440	Pv_002	K1	69.3	33.7	10.48	12.8	30.8
USDA_PI-352748	Pv_003	K2	76.3	32.1	14.91	16.5	46.0
CIAT_G11151	Pv_004	K1	63.7	22.8	13.33	17.7	57.9
USDA_PI-313888	Pv_005	K1	65.7	26.3	9.06	11.7	34.1
CIAT_G10154	Pv_006	K2	62.2	27.1	9.12	12.4	33.3
USDA_PI-151017	Pv_007	K1	63.2	27.9	10.73	14.4	38.1
USDA_PI290998	Pv_008	K2	NA	NA	NA	NA	NA
IPK_PHA3677	Pv_009	K2	49.9	28.2	13.91	23.6	48.9
CIAT_G16779	Pv_010	K2	67.1	33.6	10.75	13.6	31.7
IPK_PHA285	Pv_011	K1	74.5	36.7	12.45	14.1	33.6
USDA_PI-583642	Pv_012	K1	50.2	22.1	11.27	19.0	50.5
CIAT_G993	Pv_013	K2	65.4	42.9	10.24	13.2	23.6
CIAT_G10230	Pv_014	K1	47.2	32.5	7.69	13.8	23.4
USDA_PI-416407	Pv_015	K1	57.5	24.8	10.34	15.2	41.3
UNIBAS_58431	Pv_016	K1	53.5	26.4	13.10	20.7	49.2
UNIPG_7181	Pv_017	K2	72.3	34.8	17.36	20.3	49.4
CIAT_G1018	Pv_018	K1	51.8	25.0	6.86	11.2	27.2
IPK_PHA2134	Pv_019	K2	64.5	23.4	7.24	9.5	30.6
CIAT_G2925	Pv_020	K1	55.4	27.6	11.73	17.9	42.1
USDA_PI-309842	Pv_021	K1	58.9	24.5	10.88	15.6	44.0
UNIPG_5071	Pv_022	K2	49.2	23.8	4.76	8.2	19.8
CIAT_G17944	Pv_023	K2	57.5	29.0	11.42	16.8	39.0
UNIPG_4110	Pv_024	K1	59.5	28.3	9.24	13.1	32.3
IPK_W-18750	Pv_025	K2	64.9	39.5	16.75	21.8	42.0
UNIPG_4459	Pv_026	K1	77.0	30.1	10.15	11.1	33.4
UNIPG_3713	Pv_027	K2	58.9	31.1	14.09	20.2	44.9
IPK_PHA1684	Pv_028	K1	71.4	34.4	12.68	15.0	36.5

CIAT_G11826	Pv_029	K2	49.2	26.2	7.45	12.8	28.2
CIAT_G10110	Pv_030	K2	57.2	22.7	9.54	14.1	41.6
CIAT_G20355A	Pv_031	K1	47.8	29.9	10.13	17.9	33.6
USDA_W6-18792	Pv_032	K2	NA	NA	NA	NA	NA
IPK_PHA1916	Pv_033	K1	65.6	42.6	14.26	18.4	33.2
CIAT_G20110A	Pv_034	K1	62.4	42.8	13.05	17.7	30.2
CIAT_G20351	Pv_035	K1	71.2	31.8	11.95	14.2	37.2
CIAT_G14699	Pv_036	K1	66.6	28.2	8.25	10.5	29.0
CIAT_G900	Pv_037	K1	75.4	34.5	12.56	14.1	36.1
CIAT_G20427	Pv_038	K2	54.7	21.0	12.15	18.8	57.3
IPK_PHA1781	Pv_039	K1	44.6	30.3	7.73	14.7	25.3
CIAT_G15231	Pv_040	K1	55.3	28.8	11.17	17.1	38.4
CIAT_G8029	Pv_041	K2	45.3	32.9	14.82	27.7	44.6
USDA_PI-151014	Pv_042	K2	68.1	26.6	14.58	18.1	54.3
UNIPG_4295	Pv_043	K1	54.2	26.5	9.09	14.2	34.0
UNIPG_4458	Pv_044	K2	59.7	27.5	8.74	12.4	31.5
UNIPG_4646	Pv_045	K2	67.1	34.5	11.37	14.3	32.6
USDA_PI-152326	Pv_046	K1	47.5	21.8	7.19	12.8	32.7
USDA_PI-310586	Pv_047	K1	68.2	22.8	8.27	10.3	35.9
CIAT_G8111	Pv_048	K2	81.9	33.4	15.14	15.6	44.9
UNIBAS_VARI2	Pv_049	K2	58.0	33.8	11.23	16.4	32.9
CIAT_G18045	Pv_050	K2	51.5	26.2	12.19	20.0	46.1
UNIPG_5053	Pv_051	K2	45.3	29.9	10.86	20.3	36.0
UNIPG_5055	Pv_052	K2	51.0	29.3	10.48	17.4	35.4
CIAT_G20087	Pv_053	K1	46.5	30.9	7.39	13.4	23.7
CIAT_G10077	Pv_054	K2	55.4	25.9	9.87	15.1	37.7
CIAT_G3763	Pv_055	K1	72.4	28.6	11.93	13.9	41.3
USDA_PI-290989	Pv_056	K2	NA	NA	NA	NA	NA
CIAT_G15790	Pv_057	K2	87.9	37.5	8.78	8.4	23.2
CIAT_G10234	Pv_058	K2	58.3	28.5	9.53	13.8	33.1
NGB_NGB9299.3	Pv_059	ADM	52.5	24.5	12.82	20.7	51.8
CIAT_G15881	Pv_060	K1	59.4	30.1	9.89	14.1	32.5
CIAT_G10248A	Pv_061	K1	65.6	36.8	12.77	16.5	34.4
UNIPG_6387	Pv_062	K2	46.4	22.5	9.78	17.8	43.1
CIAT_G15443	Pv_063	K2	54.2	30.9	10.60	16.5	34.0
UNIPG_7185	Pv_064	ADM	64.9	43.0	15.15	19.7	34.9
UNIPG_4959	Pv_065	K2	64.8	30.7	9.72	12.7	31.4
UNIPG_4406	Pv_066	K1	70.1	31.6	14.89	18.0	46.7
USDA_PI-312092	Pv_067	K1	NA	NA	NA	NA	NA
CIAT_G8013A	Pv_068	K2	56.5	35.4	10.43	15.6	29.2
CIAT_G20198A	Pv_069	K1	59.0	28.4	10.43	15.0	36.4
CIAT_G3613	Pv_070	K2	52.2	32.1	13.77	22.3	42.5
CIAT_G10252	Pv_071	K1	62.3	29.3	6.93	9.4	23.4
IPK_PHA5937	Pv_072	ADM	51.9	30.6	9.39	15.3	30.4
IPK_PHA3065	Pv_073	ADM	78.9	41.9	16.41	17.6	38.8
CIAT_G10100	Pv_074	K2	56.5	33.2	11.98	17.9	35.7
UNIPG_4087	Pv_075	K2	56.9	26.5	14.35	21.3	53.6
IPK_PHA6857	Pv_076	K1	62.4	26.0	6.85	9.3	26.1
CIAT_G387	Pv_077	ADM	42.5	28.2	9.37	18.6	32.9

UNIPG_6390	Pv_078	K2	49.3	28.6	10.58	18.2	36.6
USDA_W6-17487	Pv_079	K2	NA	NA	NA	NA	NA
UNIPG_6388	Pv_080	K2	60.3	29.0	14.01	19.7	47.9
IPK_PHA130	Pv_081	K1	69.4	33.2	17.37	21.2	51.8
CIAT_G14415	Pv_082	K1	61.6	31.3	12.64	17.4	40.0
UNIPG_7587	Pv_083	K2	60.2	33.5	11.74	16.5	34.7
CIAT_G10064	Pv_084	K2	54.9	27.1	11.85	18.3	43.3
CIAT_G16741	Pv_085	K1	76.5	29.9	12.65	14.0	41.9
UNIPG_5057	Pv_086	ADM	56.9	23.3	14.09	20.9	59.9
USDA_PI-311794	Pv_087	K1	66.5	30.1	9.78	12.4	32.2
UNIBAS_Jalo EEP558	Pv_088	K2	71.9	35.8	11.81	13.9	32.7
UNIPG_5079	Pv_089	K2	52.4	23.3	11.44	18.5	48.6
CIAT_G11854	Pv_090	K1	72.0	39.0	13.26	15.6	33.7
IPK_PHA2677	Pv_091	K2	54.8	30.4	10.12	15.6	33.0
UNIPG_5058	Pv_092	ADM	65.5	32.8	15.78	20.4	47.7
USDA_PI-156669	Pv_093	K2	62.3	32.4	14.40	19.6	44.0
CIAT_G14413	Pv_094	K2	56.2	26.4	16.59	25.0	62.2
USDA_PI-290995	Pv_095	K2	51.9	34.4	9.15	14.9	26.3
UNIPG_4914	Pv_096	K1	78.5	43.6	13.21	14.2	30.0
UNIPG_5047	Pv_097	K2	52.8	30.0	13.73	22.0	45.3
UNIVPM_MIDAS	Pv_098	K2	64.6	23.4	11.02	14.4	46.7
UNIPG_5049	Pv_099	K2	73.6	33.1	11.49	13.2	34.4
UNIPG_7412	Pv_100	K2	53.3	31.1	13.29	21.1	42.3
UNIPG_4956	Pv_101	K2	53.3	28.3	9.97	15.8	34.9
IPK_PHA5913	Pv_102	K1	65.2	25.7	10.79	14.0	41.6
CIAT_G10164	Pv_103	K1	65.1	29.0	11.43	14.9	39.0
UNIPG_5069	Pv_104	K1	82.4	35.9	16.81	17.3	46.4
UNIPG_3506	Pv_105	K1	66.9	37.7	18.54	23.4	48.7
UNIPG_4554	Pv_106	K2	55.9	25.3	10.09	15.3	39.5
CIAT_G15574	Pv_107	K1	43.0	18.9	7.17	14.1	37.6
CIAT_G15930	Pv_108	K2	52.3	27.7	14.63	23.7	52.3
USDA_PI-313313	Pv_109	K1	NA	NA	NA	NA	NA
CIAT_G13765	Pv_110	K2	60.3	28.6	8.21	11.5	28.4
CIAT_G14405	Pv_111	K2	64.5	39.9	10.56	13.8	26.2
CIAT_G461	Pv_112	K1	53.5	34.3	15.74	24.9	45.5
UNIPG_4616	Pv_113	K1	78.1	30.1	19.90	21.6	65.5
CIAT_G309	Pv_114	K1	78.1	35.8	14.14	15.3	39.1
UNIPG_5084	Pv_115	K2	67.7	34.8	13.04	16.3	37.1
USDA_PI-309755	Pv_116	K1	NA	NA	NA	NA	NA
IPK_PHA7079	Pv_117	K1	86.8	27.3	14.31	13.9	51.9
UNIPG_4455	Pv_118	K2	63.3	28.5	11.74	15.7	40.8
CIAT_G15293	Pv_119	K1	57.8	34.9	10.09	14.8	28.6
USDA_PI-345574	Pv_120	K1	46.2	24.5	11.78	21.6	47.6
USDA_PI-304113	Pv_121	K1	58.0	27.4	9.26	13.5	33.5
UNIPG_7583	Pv_122	K2	84.2	37.2	16.98	17.1	45.2
CIAT_G11573	Pv_123	K1	49.7	27.6	6.97	11.9	25.0
UNIPG_4652	Pv_124	K2	59.0	26.4	14.78	21.2	55.5
USDA_PI-309831	Pv_125	K1	69.9	34.4	11.76	14.2	33.9
UNIPG_4651	Pv_126	K1	66.5	38.4	15.63	19.9	40.3

UNIPG_4362	Pv_127	K2	NA	NA	NA	NA	NA
UNIPG_3500	Pv_128	ADM	49.5	20.9	8.07	13.8	38.2
UNIPG_4361	Pv_129	K2	72.1	38.5	11.24	13.2	28.9
CIAT_G20109	Pv_130	K1	49.4	27.9	11.14	19.1	39.6
UNIPG_6389	Pv_131	ADM	45.2	39.7	16.35	30.6	40.8
CIAT_G10298	Pv_132	K2	65.9	33.9	12.82	16.5	37.5
CIAT_G15921	Pv_133	K1	51.2	27.9	15.96	26.4	56.7
UNIPG_4296	Pv_134	ADM	56.9	23.3	9.72	14.4	41.3
IPK_PHA1933	Pv_135	K1	63.5	31.4	13.89	18.5	43.8
IPK_PHA3927	Pv_136	K1	56.7	26.8	10.16	15.2	37.6
UNIPG_4299	Pv_137	K2	56.2	30.7	14.41	21.7	46.5
USDA_PI-309885	Pv_138	K1	91.1	35.6	12.60	11.7	35.1
UNIPG_4293	Pv_139	K1	54.5	27.6	8.84	13.7	31.7
UNIPG_5080	Pv_140	K1	52.0	30.6	11.03	17.9	35.7
CIAT_G10210	Pv_141	K1	58.7	27.0	9.50	13.7	34.9
CIAT_G10062	Pv_142	K2	58.1	27.5	14.41	21.0	51.9
UNIPG_7586	Pv_143	K2	65.3	36.1	11.57	15.0	31.7
IPK_PHA2198	Pv_144	K1	77.0	32.1	15.88	17.4	49.0
USDA_PI-282000	Pv_145	K2	76.9	29.2	8.98	9.9	30.5
CIAT_G20291	Pv_146	K1	89.1	33.9	15.84	15.0	46.3
USDA_PI-306149	Pv_147	K1	82.9	27.6	9.47	9.7	34.0
USDA_PI-203934	Pv_148	K1	NA	NA	NA	NA	NA
CIAT_G51731	Pv_149	K2	76.6	35.3	12.29	13.6	34.5
CIAT_G20238	Pv_150	K1	65.1	30.9	9.73	12.6	31.2
CIAT_G15856	Pv_151	K2	61.0	34.8	11.59	16.1	33.0
UNIPG_4916	Pv_152	K2	45.3	29.4	13.67	25.5	46.1
CIAT_G17830A	Pv_153	K1	58.8	21.2	16.38	23.6	76.5
CIAT_G14786	Pv_154	K2	38.4	23.4	10.75	23.7	45.5
IPK_PHA3063	Pv_155	K2	57.7	36.8	18.91	27.7	50.9
CIAT_G1029	Pv_156	K2	65.1	33.4	10.28	13.4	30.5
CIAT_G10074	Pv_157	K2	64.2	27.2	14.00	18.4	51.0
CIAT_G20259	Pv_158	K2	44.9	25.7	10.81	20.4	41.7
USDA_PI-282031	Pv_159	K2	NA	NA	NA	NA	NA
UNIPG_4294	Pv_160	K2	61.3	36.2	13.85	19.1	37.9
USDA_PI-307786	Pv_161	K1	NA	NA	NA	NA	NA
UNIPG_4102	Pv_162	K1	63.0	32.4	13.51	18.1	41.3
CIAT_G10241	Pv_163	K1	56.0	41.8	12.37	18.7	29.3
IPK_PHA7002	Pv_164	K1	88.1	38.1	16.29	15.6	42.4
USDA_PI-308891	Pv_165	K1	56.4	21.2	11.05	16.6	51.6
USDA_PI-300668	Pv_166	K1	51.5	22.1	7.25	11.9	32.5
CIAT_G15927	Pv_167	K1	60.0	30.7	9.94	14.0	32.1
CIAT_G316	Pv_168	K2	64.1	31.4	13.00	17.2	41.0
CIAT_G15618	Pv_169	K1	58.4	35.4	12.11	17.5	33.9
USDA_PI-309837	Pv_170	K1	93.7	42.6	15.55	14.0	36.2
IPK_PHA2360	Pv_171	K2	42.9	31.2	16.25	32.0	51.6
CIAT_G10112	Pv_172	K2	47.7	19.9	7.68	13.6	38.2
CIAT_G14765	Pv_173	K2	54.1	36.1	15.47	24.2	42.5
IPK_PHA358	Pv_174	K2	73.2	33.1	17.19	19.9	51.4
UNIPG_5040	Pv_175	K2	56.1	35.1	10.08	15.2	28.4

UNIPG_3722	Pv_176	K2	55.2	29.8	11.78	18.1	39.2
IPK_PHA12	Pv_177	K2	73.2	31.5	13.49	15.6	42.4
IPK_PHA1474	Pv_178	K2	77.4	42.3	14.70	16.1	34.4
IPK_PHA6266	Pv_179	K1	61.7	25.6	10.14	13.9	39.2
UNIBAS_09L0506251	Pv_180	K1	63.2	34.2	11.94	16.0	34.6
USDA_PI-151015	Pv_181	K2	80.0	39.4	15.37	16.3	38.6
USDA_PI-196927	Pv_182	K1	59.5	29.1	13.29	18.9	45.2
IPK_PHA3640	Pv_183	K1	60.3	26.6	8.55	12.0	31.8
UNIPG_3726	Pv_184	K1	71.6	36.2	15.97	18.9	43.7
UNIPG_4135	Pv_185	K2	92.3	38.1	13.79	12.6	35.9
IPK_PHA3739	Pv_186	K2	72.3	40.6	14.08	16.5	34.4
IPK_PHA2786	Pv_187	K2	43.6	28.6	9.56	18.5	33.1
USDA_W6-18758	Pv_188	K2	NA	NA	NA	NA	NA
IPK_PHA303	Pv_189	K2	59.5	30.8	7.71	11.0	24.8
NGB_NGB11752.2	Pv_190	ADM	53.3	28.5	13.90	22.1	48.3
UNIPG_7582	Pv_191	K2	NA	NA	NA	NA	NA
UNIPG_7585	Pv_192	K1	56.0	25.4	10.68	16.1	41.7

Table S2. List of SNPs associated with zinc seed content (α=0.05). Chromosome physical position, significance level ($-Log_{10}(p)$) of the detected association, phenotypic variation explained by the SNP (R^2) and Minor Allele Frequency (MAF) are reported.

**SNP**	**Chromosome**	**Position***	**$-Log_{10}(p)$**	**R^2**	**MAF**
17649_83	Pv01	49373008	5.31	0.133	C (0.47)
17621_218	Pv01	49291435	5.00	0.106	G (0.49)
17621_259	Pv01	49291394	5.00	0.106	C (0.49)
17621_228	Pv01	49291425	5.00	0.106	C (0.49)
17621_226	Pv01	49291427	5.00	0.106	C (0.49)
17598_64	Pv01	49252291	4.91	0.105	T (0.49)
17598_59	Pv01	49252296	4.91	0.105	T (0.49)
17636_40	Pv01	49330793	4.90	0.104	A (0.46)
17636_65	Pv01	49330818	4.73	0.117	G (0.49)

* *P. vulgaris* reference genome v1.0.
